# Differential Role of Axin RGS Domain Function in Wnt Signaling during Anteroposterior Patterning and Maternal Axis Formation

**DOI:** 10.1371/journal.pone.0044096

**Published:** 2012-09-05

**Authors:** Patricia N. Schneider, Diane C. Slusarski, Douglas W. Houston

**Affiliations:** Interdisciplinary Graduate Program in Genetics, Department of Biology, University of Iowa, Iowa City, Iowa, United States of America; University of Colorado, Boulder, United States of America

## Abstract

Axin is a critical component of the β-catenin destruction complex and is also necessary for Wnt signaling initiation at the level of co-receptor activation. Axin contains an RGS domain, which is similar to that of proteins that accelerate the GTPase activity of heterotrimeric Gα/Gna proteins and thereby limit the duration of active G-protein signaling. Although G-proteins are increasingly recognized as essential components of Wnt signaling, it has been unclear whether this domain of Axin might function in G-protein regulation. This study was performed to test the hypothesis that Axin RGS-Gna interactions would be required to attenuate Wnt signaling. We tested these ideas using an *axin1* genetic mutant (*masterblind*) and antisense oligo knockdowns in developing zebrafish and *Xenopus* embryos. We generated a point mutation that is predicted to reduce Axin-Gna interaction and tested for the ability of the mutant forms to rescue Axin loss-of-function function. This Axin point mutation was deficient in binding to Gna proteins in vitro, and was unable to relocalize to the plasma membrane upon Gna overexpression. We found that the Axin point mutant construct failed to rescue normal anteroposterior neural patterning in *masterblind* mutant zebrafish, suggesting a requirement for G-protein interactions in this context. We also found that the same mutant was able to rescue deficiencies in maternal *axin1* loss-of-function in *Xenopus*. These data suggest that maternal and zygotic Wnt signaling may differ in the extent of Axin regulation of G-protein signaling. We further report that expression of a membrane-localized Axin construct is sufficient to inhibit Wnt/β-catenin signaling and to promote Axin protein turnover.

## Introduction

Wnt signaling has major roles throughout development, stem cell maintenance and regeneration. Different Wnt ligand/receptor complexes can regulate distinct signaling networks that either activate the transcription factor β-catenin, or stimulate various β-catenin-independent pathways (reviewed in ref. [1]). With respect to the Wnt/β-catenin pathway, in the absence of appropriate Wnt ligands, a multiprotein complex including Axin, adenomatous polyposis coli (APC) and glycogen synthase kinase 3β (GSK3β) promotes the phosphorylation of β-catenin, resulting in subsequent recognition by ubiquitylation machinery and ultimately its degradation by the proteasome (reviewed in ref. [2]). Binding of Wnt ligands to a receptor complex, composed of Frizzled receptors (Fzd) and one of the LDL receptor-related proteins Lrp5/6, activates the Dishevelled (Dvl) protein to inhibit GSK3β and degradation complex activity. This results in unphosphorylated and stabilized β-catenin, which can enter the nucleus and complex with nuclear T-cell factor/lymphoid enhancer factor (TCF/LEF)-related transcription factors to regulate downstream target gene expression.

During early development, maternal Wnt/β-catenin signaling is required for dorsal axis induction [3]. However, following the onset of zygotic transcription, Wnt signaling is inhibited in the Organizer and anteriorly and is activated posteriorly, where it is necessary for caudalization of the body axis and nervous system [4]. The Axin protein plays a central role in the normal constitutive phosphorylation of β-catenin and is required to inhibit Wnt signaling. *Axin* (*Axin1*) was first identified as the gene affected in *fused* mutant mice, which have dorsal axis duplications and other defects [5]. Ectopic expression of Axin in dorsal blastomeres of *Xenopus* embryos caused ventralization and β-catenin destabilization [6], whereas depletion of maternal *axin1* in *Xenopus* embryos caused dorsalization and upregulation of Wnt target gene expression [7]. Additionally, *axin1/masterblind* (*mbl*) mutant zebrafish show a loss of eyes and telencephalon and other anterior defects resulting from constitutive activation of zygotic Wnt/β-catenin signaling [8], [9].

The role of Axin in cytoplasmic β-catenin destruction has been characterized extensively. In the predominant model, Axin is thought to serve as a modular scaffold to recruit GSK3β and its priming kinase, CKIα/Csnk1a1, as well as APC into a complex with β-catenin, resulting in its efficient phosphorylation, ubiquitylation and subsequent degradation (reviewed in ref. [2]). In addition to this primary role in the β-catenin destruction complex, Axin also functions in Wnt signaling initiation. Wnt stimulation results in phosphorylation of PPPSP motifs on the cytoplasmic tail of Lrp6, which bind and recruit Axin to the membrane [10], [11]. Importantly, Axin is also necessary for Lrp6 phosphorylation and is thought to facilitate PPPSP phosphorylation by bringing Gsk3β and CKI in proximity to Lrp6 [12]. This phosphorylation recruits additional Axin-Gsk3β and other signaling components to the membrane to amplify Wnt signaling. The recruitment of Axin complexes to the membrane is thought to lead to the disabling of the cytoplasmic β-catenin destruction complex.

Axin is a multi-domain protein and contains a region with high sequence and structural similarity to Regulator of G-protein Signaling proteins (RGS proteins). Prototypical RGS proteins bind to the G-alpha (Gna) subunits of heterotrimeric G-proteins, which are associated with G-protein-coupled receptors (GPCR), and accelerate their intrinsic GTPase activity. This reduces the half-life of Gna in the active GTP-bound state and negatively regulates GPCR signaling. Although the ability of Fzd to activate heterotrimeric G protein signaling has not been generally accepted, it has been shown both pharmacologically [13], [14] and genetically [15] that Fzd can function upstream of G protein signaling. Moreover, Fzd have recently been shown to act as bona fide GPCRs, intrinsically stimulating GDP-GTP exchange on Gna subunits [16], [17]. Although G-protein signaling and its regulation are increasingly recognized as critically important in Wnt signaling regulation, in vitro evidence suggests that the Axin-RGS domain lacks RGS activity. Axin can to bind to activated and unactivated Gna subunits in vitro and in transfected cells but is unable to stimulate GTP hydrolysis in single-turnover GTPase assays in vitro [18], [19], [20]. This lack of activity in vitro may reflect a true lack of GAP-like function for Axin or it may indicate that additional cofactors are required to stimulate GAP function in vivo.

The Axin-RGS domain is primarily thought to recruit APC into the β-catenin degradation complex, [6], [21], [22], where APC is essential for β-catenin ubiquitylation and degradation. Axin constructs lacking the RGS domain (AxinΔRGS) are unable to stimulate β-catenin degradation and are either inactive or act as dominant-negative proteins [5]. In agreement with these data, knock-in mice homozygous for deletion of the RGS domain show embryonic lethality and defects in β-catenin regulation [23], suggesting that the RGS domain and Axin-APC interactions are required in vivo. Owing to the importance of the Axin-APC interaction through the RGS domain, it has been difficult to determine the extent that this domain might regulate G-protein signaling in the context of Wnt/β-catenin signaling.

Here, we have investigated the role of Axin in regulating G-protein function in vivo. We generated a point mutation in a conserved residue in the RGS domain that is predicted to eliminate any GAP activity associated with Axin. Using this construct in gain and loss of function assays in *Xenopus* and zebrafish embryos, we show that Axin RGS function is likely not required for maternal Wnt signaling during axis induction, although it is necessary for zygotic function in anteroposterior nervous system patterning. These data suggest the dynamics of G-protein signaling may have different effects on Wnt signaling in different cellular contexts during development.

## Results

### Identification of a putative essential Gna-interacting residue in the Axin-RGS domain

**Figure 1:**
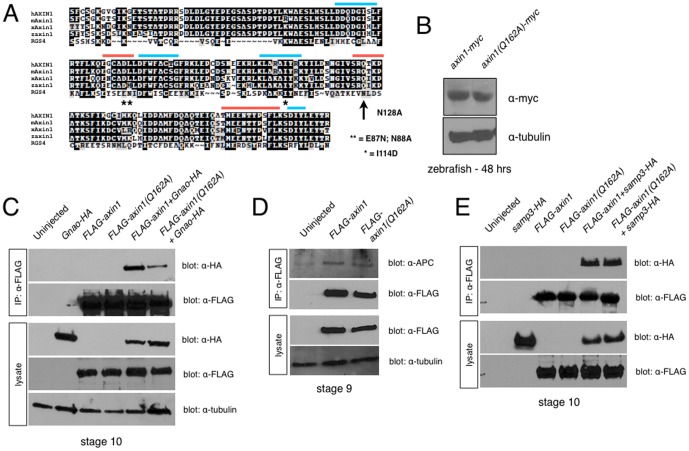
Structure-function analysis of the Axin1 RGS domain. (**A**) Alignment of RGS domains from human (hAXIN1), mouse (mAxin1), *Xenopus* (xAxin1) and zebrafish Axin1 (zAxin1) with human RGS4. Blue bars = APC binding interface; orange bars = Gna binding interface. * = residues required for GAP activity in RGS4. Arrow = residue required for RGS4 GAP activity, mutated in this study. (**B**) Immunoblots showing equivalent protein expression in 24 hpf zebrafish embryos injected with *axin1-myc* and *axin1(Q162A)-myc*. (**C**) Immunoprecipitation of FLAG-tagged Axin1 constructs with HA-tagged Gnao, showing reduced binding of FLAG-Axin1(Q162A) to overexpressed Gnao. (**D**) FLAG-Axin1 and FLAG-Axin1(Q162A) immunoprecipitate endogenous APC equivalently. (**E**) FLAG-Axin1 and FLAG-Axin1(Q162A) immunoprecipitate HA-tagged APC-SAMP3 equivalently.

To specifically address the potential role for Axin RGS activity in Wnt signaling, we sought to mutate single amino acid residues in the RGS domain that would disrupt RGS-associated GAP activity but would not affect interactions with APC, and hence not affect β-catenin degradation. Using prior mutagenesis data for RGS4 [19], [24], and the published three-dimensional structure of the Axin1-RGS domain [25], we identified several residues essential for robust GAP activity in RGS4 that are conserved (similar or identical) in zebrafish Axin1 (**Fig. 1A**). Mutation of amino acids E87, N88, I114 and N128 to non-functional residues reduced RGS4 GAP activity by over 90% [24]. We then inspected the position of these residues for proximity to the regions implicated in APC interactions and we found that residue Q162 (corresponding to N128 in RGS4) was clearly spatially separated from the APC-binding residues, whereas the corresponding E87, N88 and I114 residues were either directly adjacent (E87, N88) or within (I114) an APC-interacting fold (**Fig. 1A**). The presence of a glutamine at amino acid 128 within the Axin RGS domain is generally compatible with GAP activity. In vitro mutagenesis studies of RGS4 showed that the N128Q mutation retained significant GAP activity and binding to GTP-bound Gna proteins, although conversion to alanine (N128A) eliminated GAP activity [19], [24]. Therefore, to eliminate any potential cryptic GAP activity of the Axin RGS domain without affecting APC interactions, we generated a point mutation in zebrafish Axin1 to replace Q162 with an alanine (Q162A).

### Axin1^Q162A^ is deficient in Gna subunit interactions in vivo

In these and subsequent experiments, immunoblotting confirmed that both full-length and mutant forms of zebrafish Axin1 (Myc- or FLAG-tagged) were produced at similar levels in both zebrafish and frog embryos (**Fig. 1B** and data not shown). To evaluate the extent that the Q162A mutation might disrupt Gna interactions, we tested the ability of Axin1^Q162A^ to bind to Gna subunits in lysates of *Xenopus* embryos. We injected mRNAs encoding full-length tagged forms of Axin1 (*FLAG-axin1* or *FLAG-axin1^Q162A^*) along with *Gnao-HA* mRNAs into *Xenopus* embryos and performed coimmunoprecipitation assays. For FLAG-Axin1/Gnao-HA experiments, embryos were cultured in the crosslinker DSP prior to lysis and immunoprecipitation. Interestingly, FLAG-Axin1^Q162A^ showed reduced ability to associate with Gnao-HA in *Xenopus* embryos (**Fig. 1C**). FLAG-Axin1 could immunoprecipitate Gnao-HA, which did not associate with the immune complexes in the absence of FLAG-Axin1, suggesting a specific interaction. By contrast, FLAG- Axin1^Q162A^ pulled down about 70% less Gnao-HA.

We further confirmed that the Axin1^Q162A^ mutation did not disrupt the ability to associate with APC. Anti-FLAG-Axin1 immune complexes (from crosslinked samples) were immunoblotted with anti-APC antisera and equivalent amounts of endogenous APC were detected upon pull-down with FLAG-Axin1 and FLAG-Axin1^Q162A^ (**Fig. 1D**). Similar to these results for endogenous APC, we found no difference in the interaction of full-length or mutant Axin with an HA-tagged APC fragment containing a characterized Axin-binding motif ([25] SAMP3-HA; **Fig. 1E**). Interaction with the SAMP3 repeat was detected in the absence of crosslinkers.

**Figure 2:**
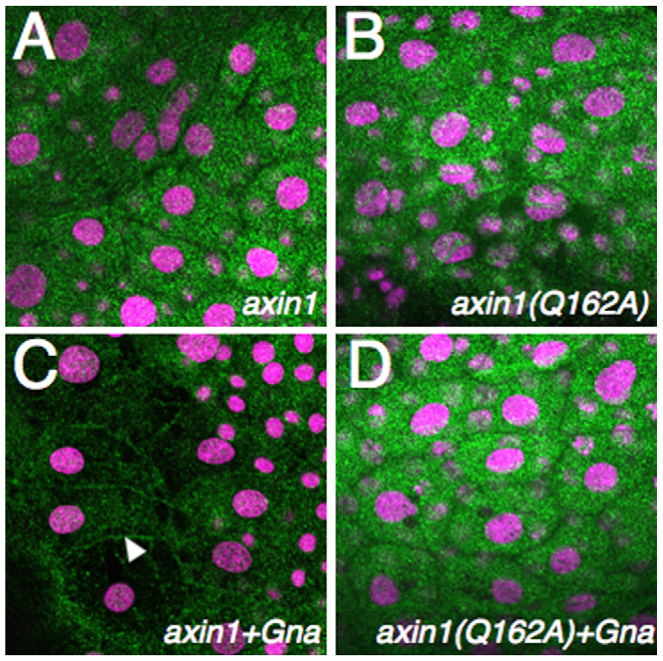
Axin1^Q162A^ does not interact with Gna at the plasma membrane. (**A–D**) Immunostaining against Myc showing localization of Axin1-Myc and Axin1(Q162A)-myc in zebrafish embryos, without (A, B) or with coexpression of Gnao (C, D). The arrowhead in C indicates membrane localization of Axin1 upon Gnao expression. Embryos were counterstained with TOPRO3 to show nuclei (purple).

Since the ability of the Axin-RGS domain to alter the GTP hydrolysis properties of Gna proteins is not measurable in vitro [18], [20], it was unclear whether this reduced binding reflected loss of bona fide RGS activity. In *Drosophila*, activation of Gna is sufficient to recruit Axin to the plasma membrane [26]. We therefore injected mRNAs encoding Myc-tagged Axin1 and Axin1^Q162A^ in the presence or absence of Gnao in zebrafish embryos and ascertained the degree of Axin membrane localization. Embryos were fixed at the epiboly stages and immunostained for the Myc epitope. Myc-Axin1 and Myc-Axin1^Q162A^ were cytoplasmic and distributed in numerous puncta within cells of the superficial epithelial enveloping layer (EVL) as well as deep cells (**Fig. 2A, B**). In embryos coinjected with *gnao* mRNA, Axin1 was redistributed away from the cytoplasm to the plasma membrane in the EVL, whereas Axin1^Q162A^ remained expressed in cytoplasmic puncta (**Fig. 2C, D**). Overall, these data show that glutamine 162 in the Axin1 RGS domain is analogous to a conserved residue needed for function in other RGS domains and is necessary for the proper interaction of Axin with Gna proteins.

### Axin1-Gna interactions are not required for maternal Wnt signaling

**Figure 3:**
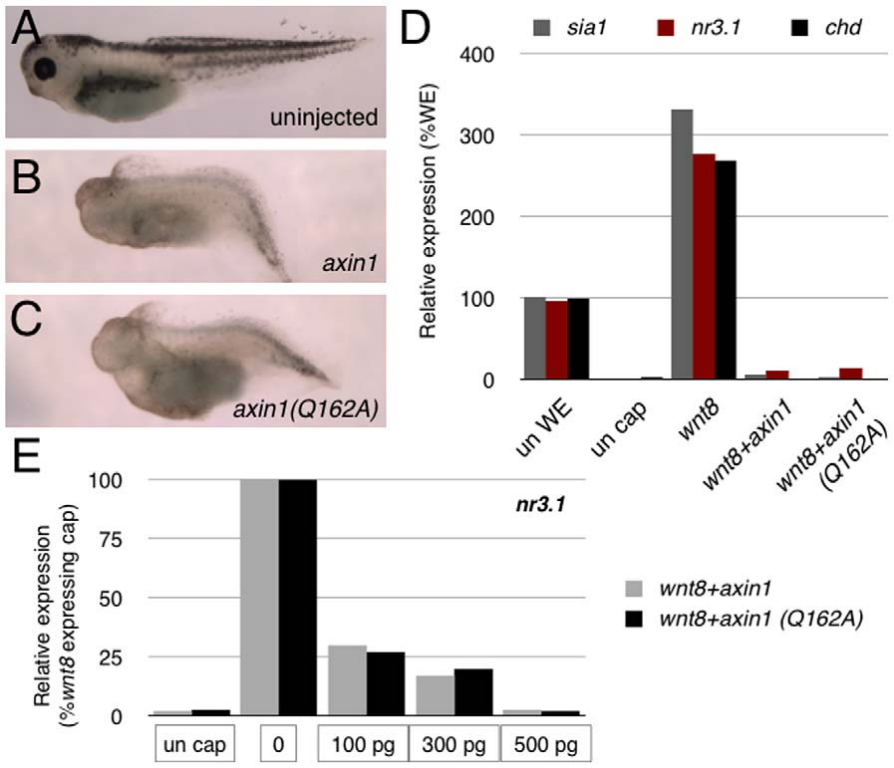
Overexpression of Axin1^Q162A^ blocks axis formation and Wnt signaling in *Xenopus* embryos. (**A–C**) Representative phenotypes of stage 40 control uninjected embryos (**A**), *axin1*-injected embryos (500 pg), (**B**) and *axin1(Q162A)*-injected embryos (500 pg) (**C**). (**D**) Representative realtime RT-PCR of *sia1*, *nr3.1* and *chd* expression in control whole embryos (un WE), control animal caps (un cap) and caps injected with *wnt8* (1 pg) and Axin constructs (500 pg). (**E**) Realtime RT-PCR of *nr3.1* expression in animal caps injected with *wnt8* and different doses of Axin constructs (100 pg, 300 pg, 500 pg; indicated below sets of bars).

To test the extent that Axin1^Q162A^ could function normally in Wnt/β-catenin signaling, we overexpressed mRNAs encoding zebrafish Axin1 and Axin1^Q162A^ in *Xenopus* embryos. Injection of full-length *axin1* caused ventralization, similar to published reports [5]. Overexpression of *axin1^Q162A^* also ventralized embryos to a similar extent, resulting in loss of head structures (brain and eyes) and enlargement of ventral posterior tissues (**Fig. 3A–C**). To examine whether these effects were directly related to the inhibition of Wnt/β-catenin signaling, we performed animal cap assays. Caps were cut from late blastulae previously injected with *wnt8* mRNA in the presence or absence of either *axin1* or *axin1^Q162A^* and expression of direct Wnt target genes relevant for dorsoventral patterning (*Xenopus siamois (sia1)*, *nodal-related 3 (nr3.1)* and *chordin (chd)*, [27], [28], [29] was analyzed by RT-PCR. Consistent with our observations on whole embryos, both forms of Axin1 were sufficient to block the activation of target genes by injected *wnt8* (**Fig. 3D**). Additionally, both forms showed a similar dose response of *wnt8* inhibition in animal caps, when injected over a range of mRNA amounts in the same experiment (**Fig. 3E**). These data show that Axin^Q162A^ is sufficient to inhibit Wnt signaling when overexpressed and suggest that the Q162A mutation does not affect the overall ability of Axin to inhibit Wnt/β-catenin activity. Similar inhibitory effects on axis formation were seen in zebrafish embryos upon overexpression of higher doses of *axin1^Q162A^* (data not shown).

Since both full-length and mutant Axin overexpression could inhibit endogenous primary axis formation, we suspected that Axin-Gna interactions would not be required in this context. However, in these experiments, Axin1 was greatly overexpressed, whereas Axin is normally considered limiting in Wnt signaling [30]. We therefore performed rescue assays as a more stringent test of Axin1 RGS domain function during axis formation. Maternal Wnt signaling is required for *Xenopus* dorsoventral axis formation [31] and depletion of maternal *axin1* mRNA in *Xenopus* results in elevated Wnt/β-catenin activity and dorsalization of the embryo [7]. To test whether Axin1^Q162A^ could rescue Axin loss-of-function in this context, we injected antisense oligonucleotides against *axin1* into stage VI oocytes, followed 24 hours later by injection of either zebrafish *axin1* or *axin1^Q162A^* mRNAs. We used an mRNA dose of 60 pg, which had no effect on dorsoventral patterning when injected into control oocytes and which was most effective in previous rescue experiments (data not shown and Ref [7]). Embryos were derived from oligo-injected and rescued oocytes by transferring them into an egg-laying host female for in vitro fertilization [32]. The resulting embryos were either frozen down at the gastrula stage for RT-PCR analysis or left to develop for morphological and gene expression analysis at the tailbud stage.

**Figure 4:**
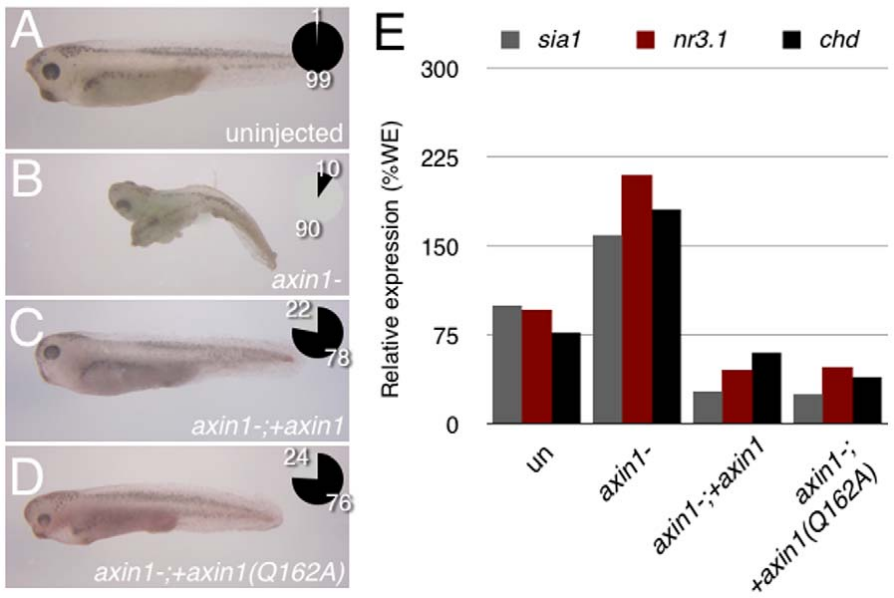
Both *axin1* and *axin1(Q162A)* rescue hyperdorsalization in maternal *axin1*-depleted embryos. (**A–D**) Representative phenotypes of control and *axin1*-depleted *Xenopus* embryos obtained following host transfer. Chart showing distribution of phenotypes is inset in each panel, percentages are indicated; black = normal, grey = dorsalized. (**A**) control uninjected stage 37 embryo, (n = 40) (**B**) *axin1*-depleted embryo (4 ng oligo; n = 44), (**C**) *axin1*-depleted embryo injected with 60 pg *axin1* mRNA (n = 32), (**D**) *axin1*-depleted embryo injected with 60 pg *axin1(Q162A)* mRNA (n = 21). (**E**) Representative realtime RT-PCR of *sia1*, *nr3.1* and *chd* expression in control whole embryos (un), *axin1-*depleted embryos (*axin-*) and *axin-* embryos injected with *axin* constructs.

*axin1*-depleted embryos displayed a dorsalized appearance, in a majority of cases developing second axes or enlarged heads and cement glands (90%; n = 44; **Fig. 4**). Intriguingly, injection of either *axin1* or *axin1^Q162A^* into *axin1*-depleted oocytes partially rescued the maternal knockdown of *axin1*, reducing the incidence of dorsalized embryos to 22% (n = 32) and 24% (n = 21) respectively (**Fig. 4**). To examine the effects of maternal *axin1* depletion and rescue on Wnt-dependent transcription, we analyzed levels of Wnt targets *sia1*, *nr3.1* and *chd.* Depletion of maternal *axin1* resulted in hyperactivation of these genes, as assessed by RT-PCR (**Fig. 4E**). Consistent with our phenotypic data, injection of *axin1* and *axin1^Q162A^* could both reduce expression of both genes to near-normal levels. A representative analysis is shown in **Fig. 4**; results were repeated three times with similar results. Taken together with the overexpression data, these results suggest that Axin RGS-Gna interactions are likely not necessary in the regulation of maternal Wnt signaling during *Xenopus* dorsoventral axis specification.

### Axin1-Gna interactions are required for zygotic Wnt signaling in anterior brain patterning

We additionally studied the role of Axin RGS domain-Gna interactions in zygotic Wnt/β-catenin signaling using a zebrafish genetic mutant, *masterblind* (*mbl*), which encodes an *axin1* loss-of-function allele [8], [9]. The *mbl* mutation was originally identified in a screen for genes involved in forebrain development [33]. Homozygotes display absent or reduced telencephalon, absence of olfactory placodes and optic vesicles, and an expanded diencephalon, suggesting a role for Axin1 in restricting Wnt/β-catenin activity in the anterior nervous system [33].

**Figure 5:**
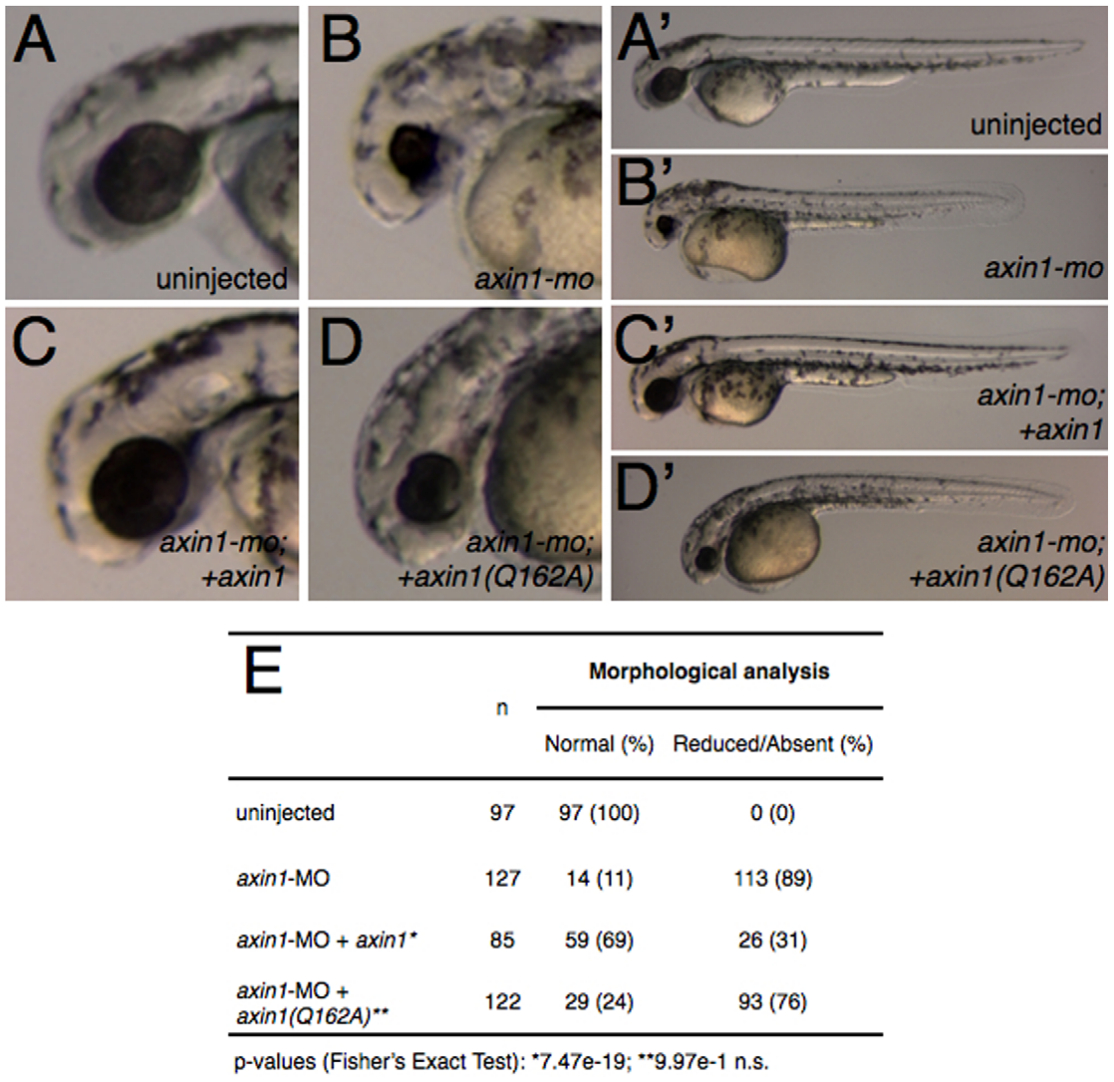
*axin1* but not *axin1(Q162A)* rescues anterior defects in Axin1-depleted embryos. (**A–D′**) Representative phenotypes of control and Axin1-depleted zebrafish embryos. (**A**) control uninjected embryo, (**B**) Axin1-depleted embryo (4 ng *axin1*-MO), (**C**) MO injected embryo coinjected with 25 pg *axin1* mRNA, (**D**) MO injected embryo coinjected with 25 pg *axin1(Q162A)* mRNA. (**E**) Summary table of morphological defects.

**Figure 6:**
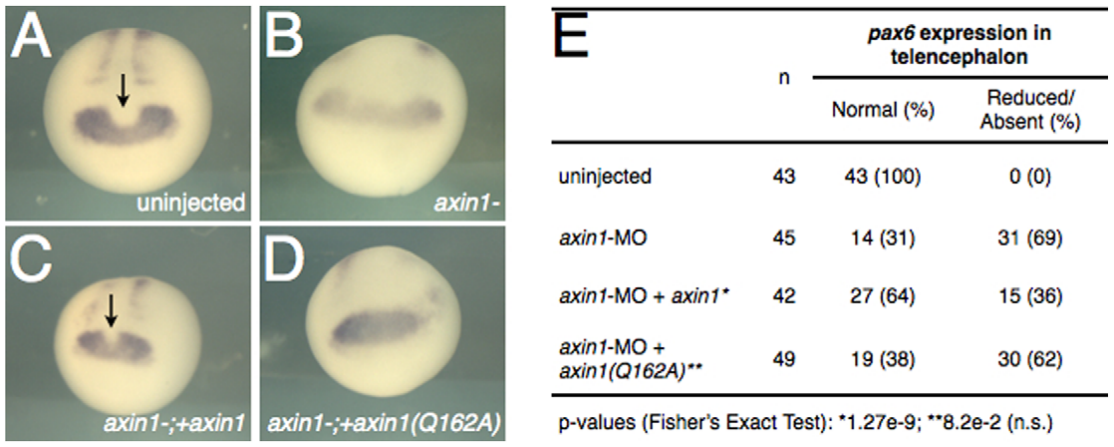
*axin1* but not *axin1(Q162A)* rescues telencephalic defects in Axin-depleted embryos. (**A–D**) Representative examples of *pax6* expression in control and Axin1-depleted *Xenopus* embryos. Arrows indicate the presumptive telencephalon region of the eye field, visible as a narrowed medial notch in the area of *pax6* expression. (**A**) Control uninjected stage 20 embryo, (**B**) Axin1-depleted embryo (20 ng *axin1*-MO), (**C**) MO injected embryo coinjected with 60 pg *axin1* mRNA, (**D**) MO injected embryo coinjected with 60 pg *axin1(Q162A)* mRNA. (**E**) Summary table of *pax6* expression data.

**Figure 7:**
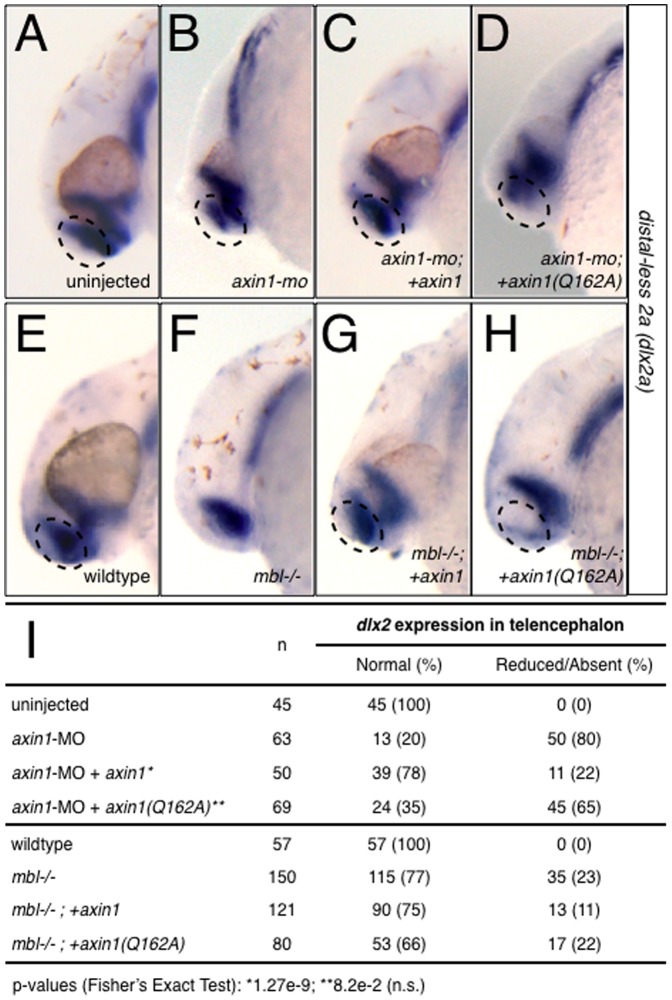
*axin1* but not *axin1(Q162A)* rescues telencephalic defects in Axin1-depleted and in *mbl*−/− embryos. (**A–H**) Representative examples of *dlx2* expression in control and Axin1-depleted zebrafish embryos (A–D) and in wildtype and *mbl−/−* embryos (E–H). (**A**) control uninjected embryo at 24 hpf, (**B**) *axin1-*MO-injected, (**C**) *axin1*-MO+xx *axin1*, (**D**) *axin1-*MO+*axin1(Q162A)*, (**E**) wildtype embryo at 24 hpf, (**F**) *mbl−/−*, (**G**) *mbl−/−;+axin1* (**H**) *mbl−/−;+axin1(Q162A)*. (**I**) Summary of *dlx2* expression data. Arrows indicate areas of reduced or absent *dlx2* in the telencephalon.

We first performed a series of experiments using an antisense morpholino phosphorodiamidate oligonucleotide (morpholino, MO) against *axin1* in zebrafish to phenocopy *mbl* [33]. Injection of *axin1*-MO into 1–2 cell embryos resulted in a high percentage of embryos with phenotypes identical to the *mbl* mutant (89%; n = 127), namely reduced telencephalon and reduced or absent eyes (**Fig. 5**). Similar results were seen in frog embryos (see below, **Fig. 6**). These phenotypes were assessed both by morphology at 28 hours-postfertilization (hpf) and by in situ hybridization at the 24 somite stage against *distal-less homeobox gene 2a* (*dlx2a*), a marker of ventral telencephalon, diencephalon and pharyngeal arches [34]. Embryos injected with *axin1*-MO showed reduced *dlx2a* expression in the telencephalon in the majority of cases (**Fig. 7B**; 80%; n = 63), whereas diencephalon and pharyngeal arch expression was unaffected.

We next compared these phenotypes with those of embryos coinjected with either *axin1* or *axin1^Q162A^* along with the MO in rescue experiments. Injection of full-length *axin1* mRNA partially rescued the MO phenotype, resulting in substantially fewer embryos showing reduced telencephalon (31%; n = 85; **Fig. 5**) and telencephalic *dlx2a* expression (22%; n = 50; **Fig. 7C**). By contrast, *axin1^Q162A^* did not rescue the MO-induced telencephalic defects, resulting in similar numbers of embryos with morphologically reduced/absent telencephalon (76%; n = 122; **Fig. 5**) and reduced *dlx2a* expression (65%; n = 69; **Fig. 7D**).

MO depletion experiments in *Xenopus* also showed that *axin1* and *axin1^Q162A^* differed in their ability to rescue inhibition of zygotic Axin1 function (**Fig. 6**). In this case, *pax6* was used as a marker of telencephalon and diencephalon, with a distinctive notch forming in the medial expression domain in the position of the telencephalon [35]. This medial domain was absent in embryos injected with MO against *Xenopus axin1* and could be rescued by *axin1* but not by *axin1^Q162A^*. These data show that Axin RGS-Gna interactions are necessary to fully restore Axin function during anteroposterior patterning of the CNS.

To more conclusively demonstrate the requirement for Axin RGS-Gna interactions in anteroposterior patterning, we performed a separate series of experiments in *mbl* mutants, which are genetically deficient in Axin1 activity. We injected zebrafish *axin1* or *axin1^Q162A^* mRNAs into progeny obtained from crosses of *mbl* heterozygotes. Such a cross results in ∼25% homozygous mutant offspring, which display defects in anteroposterior brain patterning. Fertilized eggs from a mating of *mbl* heterozygotes were injected at 1–4 cell stage with wildtype *axin1* or *axin1^Q162A^* mRNAs and scored for head defects at 24 hpf and by morphology and *dlx2a* expression at 30 hpf as above. Both constructs produced similar steady-state protein levels in zebrafish embryos (**Fig. 1**).

Uninjected embryos from *mbl* heterozygote crosses showed the predicted frequency of mutant phenotypes (23%; n = 150; **Fig. 7F**). Genomic DNA sequencing of affected fish, but not wildtype-looking fish, showed the presence of the expected T-to-A transversion at nucleotide 1220 [8]. Injection of *axin1* mRNA was able to reduce the incidence of *mbl* phenotypes (11%; n = 121), whereas embryos injected with *axin1^Q162A^* had a similar incidence of mutant phenotypes as the uninjected samples (22%; n = 80; **Fig. 7G, H**). We sequenced the *axin1* genomic locus from five embryos with normal phenotypes generated from crosses injected with *axin1*. Sequencing confirmed that three were homozygous for the *mbl* point mutation (data not shown), indicating that the normal phenotypes in those cases were indeed the result of rescue of mutant embryos.

Overall, these data suggest that Axin-RGS domain function, at the level of Gna interaction, is required to attenuate normal Wnt signaling during anteroposterior patterning of the brain.

### Membrane localization of Axin is sufficient for maternal Wnt signaling and for Axin protein turnover

**Figure 8:**
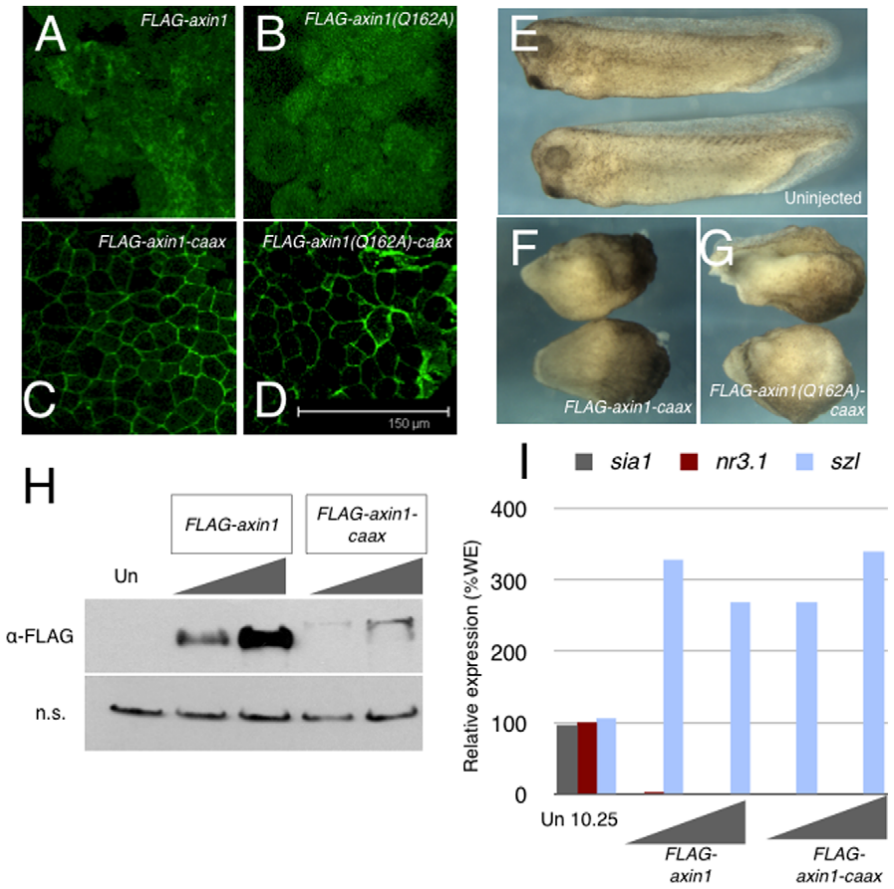
CAAX-tagged Axin1 is sufficient to inhibit axis formation and to promote Axin1 protein turnover. (**A–D**) Localization of CAAX-tagged and untagged Axin1 proteins in *Xenopus* animal caps. (**E–G**) Uninjected control embryos (**E**) and embryos expressing Axin1-CAAX (**F**) and Axin1(Q162A)-CAAX (**G**) at the tailbud stage. (**H**) Immunoblots of stage 9 embryo lysates injected with *FLAG-axin1* (100 pg, 300 pg) and *FLAG-axin1-caax* (100 pg, 300 pg). The top panel shows anti-FLAG blotting, the bottom panel shows a non-specific band (n.s.) detected by the anti-FLAG antibody to confirm equivalent loading. (**I**) Representative realtime RT-PCR of *sia1*, *nr3.1* and *sizzled (szl)* expression in control embryos (un) and in embryos injected with *FLAG-axin1* (100 pg, 300 pg) or *FLAG-axin1-caax* (100 pg, 300 pg).

Axin-Gna binding could be necessary to regulate the turnover of Gna-GTP, as is typical of canonical RGS proteins. Although this activity has never been demonstrated for Axin in vitro, it may exist in vivo. Alternatively, Axin membrane recruitment mediated by Gna interaction could be needed for some aspect of Wnt inhibition, possibly for the assembly of β-catenin degradation complexes. Axin membrane localization is also required for Wnt signaling activation and has been implicated in regulating Axin degradation. To determine the extent that Axin1 membrane localization by Gna activation is sufficient for its function in Wnt signaling, we generated membrane localized FLAG-Axin1 and FLAG-Axin1^Q162A^ by tagging with the CAAX prenylation sequence at the C-terminus. We confirmed that both constructs were expressed and localized to the membrane by immunostaining animal caps from injected *Xenopus* embryos for the FLAG epitope (**Fig. 8A–D**). Additionally, both *FLAG-axin1-caax* and *FLAG-axin1^Q162A^* could ventralize embryos when overexpressed (**Fig. 8E–G**). Unfortunately, we found that the steady-state levels of Axin were severely compromised by the addition of the CAAX sequence. In an initial series of *axin-MO* rescue experiments in zebrafish, injection of high doses of either CAAX-tagged construct caused primary axis defects (data not shown), showing they were active in fish. However, injection of lower non-ventralizing doses (∼20 pg) failed to rescue the effects of *axin1*-MO injection. We analyzed the perdurance of the protein by immunoblotting and we were unable to detect the presence of either CAAX construct at non-ventralizing doses (data not shown).

We further compared the steady state levels of Axin and Axin-CAAX in *Xenopus* embryos. Injection of 100 pg or 300 pg of *FLAG-axin1* or *FLAG-axin1-caax* effectively ventralized embryos (**Fig. 8**) although *FLAG-axin1-caax* produced substantially less protein in lysates of late blastulae (**Fig. 8H**). Thus, membrane localization is sufficient to inhibit maternal Wnt signaling and for Axin turnover. However, since the doses needed to produce detectable protein by the gastrula stage strongly ventralize the embryo, it thus was not possible to study the role of Axin membrane localization in zygotic Wnt signaling by mRNA injection. We attempted to overcome this problem using DNA injections in *Xenopus* embryos, but even low doses of *FLAG-axin1-caax* plasmid DNA (5–10 pg) caused gastrulation abnormalities (data not shown). Although these data suggest that Axin membrane localization is sufficient to inhibit Wnt signaling in the early embryo, it remains to be determined whether this is sufficient for anterior brain pattering as well.

## Discussion

The presence of the RGS domain in Axin has suggested that interaction with heterotrimeric G-proteins through this domain could be an important step in regulating the activity of the β-catenin degradation complex. To specifically address the role of Gna interactions in Axin function, we made a point mutation in the Axin-RGS domain to inhibit Gna interactions while leaving Axin's APC interactions and functions in the β-catenin degradation complex intact. Overall, we showed that Axin1^Q162A^ failed to interact normally with Gna and was able to rescue maternal Axin loss-of-function, but not zygotic Axin defects during anterior brain patterning. Furthermore, we find that Axin membrane localization is sufficient for its maternal function and for Axin protein turnover. These data suggest that regulation of Wnt signaling is highly context dependent, which may reflect differing mechanisms of Wnt activation or morphogen gradient interpretation.

### Role of Axin in Fzd/GPCR signaling

Although the idea that Fzds are indeed GPCRs is gaining acceptance, reviewed in Ref. [36], the mechanisms of interaction with the β-catenin degradation machinery has remained unclear. Several groups have recently investigated the mechanisms of heterotrimeric G-proteins in Wnt signal transduction. Gna protein activation is sufficient to disrupt β-catenin degradation complexes [37] and to recruit Axin to the plasma membrane [26]. Activated Gna is thought to primarily antagonize the function of Axin in the degradation complex through the RGS domain [26]. We might therefore expect that an Axin construct unable to be recruited by Gna should be competent to repress Wnt signaling. Although our results suggest this is the case for maternal Wnt signaling, we found that Axin deficient in Gna interaction cannot repress anterior nervous system Wnt signaling.

Our results provide two possible insights into the role of Axin-G-protein interactions in Wnt signaling. First, if indeed Axin-RGS possesses cryptic GAP activity in vivo, Axin could increase the rate of GTP hydrolysis by Gna, in a manner consistent with prototypical RGS proteins. It is possible that Axin-RGS exhibits GAP activity in vivo when associated with other cofactors, although demonstrating this idea is likely to require developing new methods to study GTPase activity in living cells. Additionally, Axin without the ability to interact with and shutdown Gna-proteins should still be expected to function in the cytoplasmic β-catenin degradation complex. Gna may therefore have as yet undetermined roles in promoting Wnt signaling. Increased Gna GTP hydrolysis could also promote the reassociation and inactivation of Gnb/Gng (beta/gamma) subunits, which have been implicated in regulating Wnt activity through Dvl function and Lrp6 phosphorylation [26], [38].

A second prediction from our data is that Axin membrane localization might be necessary for its ability to reassemble β-catenin degradation complexes following Wnt stimulation. Axin is recruited to the membrane to initiate receptor signaling [10], [12]. Although Axin degradation is a long-term result of membrane localization [10], [39], a subset of Axin must dissociate from this membrane complex to rapidly reassemble cytoplasmic β-catenin degradation complexes. Axin phosphorylation is known to promote degradation complex assembly and Axin stability [39] and Axin could be phosphorylated at the membrane by GSK3β or other kinases. We speculate that this recycling of Axin would be most critical in instances where cells experience subtle differences in Wnt gradients, such as in the reiterated use of Wnt signaling in establishing posterior subdomains in the nervous system. In this case, the synthesis of new Axin protein from mRNA may be too slow to sufficiently reestablish baseline β-catenin degradation. Very little is known about the kinetics of Axin translation.

### Role of Axin in maternal versus zygotic Wnt regulation

Since Axin1^Q162A^ showed reduced Gna interaction, it might therefore be expected to function as a constitutive repressor. This was not the case, since we found that Axin1^Q162A^ was unable to repress elevated Wnt signaling when expressed in the *masterblind* fish. By contrast, we find that Axin1^Q162A^ is able to rescue maternal *axin* depletion in *Xenopus*, suggesting that Gna association may be less important in this instance. The differences we see between the role of Axin in neural patterning and in axis formation could be explained by differences in receptor composition or presence of additional cell type-specific Wnt regulators. G-protein signaling may not be important for maternal Wnt signaling in *Xenopus* since there is suggestion, albeit a controversial one, that β-catenin may be stabilized intracellularly, independent of membrane receptors, reviewed in Ref. [40]. Other evidence suggests that *Xenopus* Wnt11b and Wnt5 can mediate axis induction and β-catenin stabilization [41]. The mechanisms regulated by Wnt11/5 are not known, and it is possible that signaling by these Wnts in this context is less dependent on G-protein signaling.

An alternative explanation is that cells undergoing neural patterning must interpret slight differences in Wnt concentrations along the anteroposterior axis over time, and thus require rapid shutdown of Wnt receptor activity. In the case of axis induction, more of an all-or-nothing response would be involved and the persistence of receptor signaling would be less critical. Unfortunately, we were unable to test the sufficiency of membrane localization for zygotic Wnt regulation, owing to the rapid turnover of Axin-CAAX and the side effects of Axin-CAAX expression from plasmids on gastrulation. In this latter case, it is possible that the ability of Axin to activate JNK signaling [42], and thus disrupt convergent extension movements, is stimulated by constitutive membrane localization in the gastrula. Transgenic approaches to express Axin-CAAX in the prospective telencephalon will be necessary to address this question.

Overall, our data suggest that interactions of Gna with the Axin-RGS domain are critical in Wnt morphogen gradient interpretation in vivo, but may be less important in other contexts. These studies indicate the importance of dissecting Wnt in vivo in different contexts, since many studies in cell lines and overexpression settings cannot recapitulate endogenous morphogen gradients.

## Materials and Methods

### Ethics Statement

All animal protocols used in these studies were approved by the Institutional Animal Care and Use Committee at The University of Iowa.

### Danio rerio embryo manipulation

Fertilized eggs were collected from natural spawning of adult zebrafish. Embryos were rinsed with embryo medium [43] and raised at 28°C. Staging was done according to Ref. [44]. *Masterblind* (*axin1^tm213/+^* (AB)) embryos were obtained from the Zebrafish International Resource Center (ZIRC) and propagated for use in this study. Homozygous embryos were generated by pairwise in-crosses of heterozygous adult carriers.

### Xenopus laevis embryo manipulation

Eggs were recovered from hCG-stimulated (1000 U) females, fertilized using a sperm suspension and maintained in 0.1× MMR [45]. Embryos were dejellied in 2% cysteine in 0.1× MMR, pH 7.8, washed extensively in 0.1× MMR and transferred to 2% Ficoll 400 in 0.5×MMR prior to injection. *Axin1* mRNA, encoding the wildtype and mutant forms, was injected with or without *wnt8* mRNA, animally at the 2–4 cell stage (500 pg/embryo). Several hours after injection, embryos were transferred to 0.1×MMR and cultured to the desired stage. Staging was done according to Nieuwkoop and Faber [46].

For animal cap assays, embryos were injected and cultured to the late blastula stage. Embryos were transferred to agarose-coated dishes in 1× MMR, and animal caps were dissected using sharp forceps following removal of the vitelline membrane. Caps were cultured several hours and then frozen for analysis.

### Plasmids and mRNAs

The coding regions (CDS) for zebrafish *axin1*, *gnai* and *gnao* were amplified by PCR from zebrafish embryo cDNA (24 hpf) and cloned into pCR8/GW/TOPO (Invitrogen). A 459 b.p. DNA fragment encompassing the SAMP3 repeat (153 a.a.) of Xenopus *apc* (n.t. 5842–6300) was synthesized (gblock assembly, Integrated DNA Technologies (IDT)) and cloned into pCR8/GW/TOPO. Sequences were verified and selected clones in the 5′-L1 to 3′-L2 orientation were recombined into Gateway compatible vectors: pCS2+, pCS2+HA, pCS2+FLAG or pCS3-Myc (custom vector conversion kit; Invitrogen). Details of the Gateway plasmids are available upon request. Mutagenesis was performed using the QuickChange kit according to the manufacturer's instructions (Stratagene); mutagenic primers were designed using PrimerX (http://www.bioinformatics.org/primerx). Template DNAs for in vitro transcription were prepared by NotI digestion and capped messenger RNA was synthesized using SP6 mMESSAGE mMACHINE Kits (Ambion).

### Antisense oligonucleotides

*Xenopus axin*-M7: T*T*C*CTCGCCAGGAA*C*T*G*G; Ref. [7]

*Xenopus axin1*-MO: ACTCATTTTGGTGGCGTTTGTATAC; (n.t. 248-72; new)

Zebrafish *axin1-*MO: ACTCATGCTCATAGTGTCCCTGCAC; Ref. [47]

(* = phosphorothioate bond; start codons are underlined). *axin*-M7 (HPLC purified) was resuspended in sterile filtered water to 1 mM and stored in aliquots at −80°C. Morpholino oligos (MOs) were obtained from Gene Tools, LLC, resuspended in water to 1 mM and stored at 4°C.

### Whole mount in situ hybridization

Whole-mount in situ hybridization on *Xenopus* and zebrafish embryos was performed as described [47]. Template DNAs for probe synthesis were prepared by restriction digestion and in vitro transcription, using the designated enzymes and polymerases: *D. rerio dlx2a* (*Bam*H1/T7; a gift from Dr. Robert Cornell, The University of Iowa, Iowa City, IA USA) and *Xenopus pax6* (*Eco*R1/T7). Probes were labeled during synthesis with digoxigenin-11-UTP (Roche), using polymerases and reaction buffers from Promega. After detection using BM Purple (Roche Applied Science), embryos were refixed, mounted and photographed. *Xenopus* embryos were bleached prior to imaging.

### RT-PCR

Total RNA from embryos and explants was prepared and cDNA was synthesized as described [48]. Real time PCR was carried out on the LightCycler 480 system (Roche Applied Science) according to the manufacturer's instructions. Products were detected using SYBR green. Values were normalized against *ornithine decarboxylase* and expression values were determined by comparing against a standard curve of serially diluted control embryo cDNA. Experiments were repeated at least three times using embryos from different females, although the data shown are representative individual experiments. Detailed protocols and primer sequences are available upon request.

### Host transfer experiments

For the *axin1* maternal depletion, full-grown stage VI oocytes were manually defolliculated and oligos targeting *axin1* were injected into the vegetal pole (4 ng per oocyte) as described previously [7]. Oocytes were cultured for 24–30 hours at 18°C, after which full length or mutant *axin1* mRNAs (60 pg) were injected and the oocytes were stimulated to mature by the addition of progesterone (2 µM final concentration). After 12 hours, the mature oocytes were colored with vital dyes and surgically transferred into an egg-laying host female as described [32]. After ∼3 hours, the female was squeezed and the eggs were fertilized with a sperm suspension. After removal of the jelly coats, cleaving colored embryos were sorted from host embryos and raised to the desired stages in 0.1× MMR.

### Western blot

Zebrafish embryos were lysed in phosphoprotein buffer (80 mM ß-glycerophosphate pH 7.0, 20 mM EGTA, 15 mM MgCl_2_, 1 mM DTT, 1 mM PMSF, 1∶50 protease inhibitor cocktail; Sigma) and cleared by centrifugation at 15, 000×g. The equivalent of 10 embryos were loaded on 10% SDS-PAGE Ready Gels (BioRad) and transferred to nitrocellulose. Membranes were blocked in 5% non-fat dry milk in PBS+0.1% Tween 20 and incubated in primary antibody (mouse anti-myc 9E10 (DSHB); 1∶1000 dilution of concentrate) diluted in the same buffer. Detection was performed using the Super Signal West Pico system (Thermo/Pierce). Exposure times were approximately one minute.

### Immunostaining

Zebrafish embryos were fixed in cold 4% paraformaldehyde in 1× PBS for 1 hour, washed in PBS and blocked in PBS+10% goat serum. Samples were incubated overnight with anti-Myc monoclonal antibody (1∶500 of concentrate, mAb 9E10, DSHB) followed by secondary antibody conjugated with fluorescent secondary antibodies (goat anti-mouse Alexa-0488, Invitrogen/Molecular Probes), as described in Ref. [49]. Embryos were examined using confocal microscopy (63× PL APO 1.2 NA water immersion objective; Leica). For *Xenopus* animal caps, dissection was done on agarose-coated dishes in low-calcium/magnesium MMR, to slow healing. The explants were fixed for 20 minutes in MEMFA and washed three times in PBS/0.1% Tween. Blocking was done as above and samples were incubated in anti-FLAG M2 (1∶1000).

### Coimmunoprecipitation

*Xenopus* embryos were coinjected at the 4-cell stage with *gnao-HA* or *samp3-HA* and *FLAG-axin1* or *FLAG-axin1^Q162A^* and cultured to stage 10. To detect Axin-Gnao interactions, samples were crosslinked in vivo using 1 mM DSP for 30 minutes (Dithiobis(succinimidyl) propionate; Thermo/Pierce). Samples rinsed in buffer containing 20 mM Tris and lysed in ice-cold lysis buffer (150 mM NaCl, 20 mM Tris-HCl, 1 mM EDTA, 1 mM EGTA, 1% Triton X-100, containing Halt Protease inhibitor cocktail; Thermo/Pierce). The lysates were then incubated for 2 hours at 4°C with 2 µg mouse anti-FLAG M2 mAb (clone M2; Sigma) and then added to protein A/G beads (20 µl bed volume; Thermo/Pierce) and rocked for another 2 hours. The immune complexes were washed three times with lysis buffer, resuspended in 20 µl SDS-PAGE buffer and subjected to SDS-PAGE and transfer as above. Membranes were blocked in non-fat dry milk in PBS-0.1% Tween-20 and immunoblotted against FLAG (mouse anti-FLAG M2; 1∶1000) and against HA (rat anti-HA clone 3F10, 1∶500, Roche Applied Science).
